# Presence of t(14;18) translocation in healthy individuals varies according to ethnic background in the Brazilian population

**DOI:** 10.1590/1414-431X20176172

**Published:** 2017-06-05

**Authors:** D. Levy, E.R.M. Bertoldi, J.L.M. Ruiz, J. Pereira, S.P. Bydlowski

**Affiliations:** 1 Laboratório de Genética e Hematologia Molecular (LIM31), Faculdade de Medicina, Universidade de São Paulo, São Paulo, SP, Brasil; 2 Universidade Federal da Integracão Latino-Americana, Porto Belo, Foz do Iguaçu, PR, Brasil

**Keywords:** t(14;18), Lymphomas, Characterization, Molecular genetics

## Abstract

Several groups have demonstrated that healthy individuals can present the t(14;18) translocation. In this report, the presence of the translocation was examined in healthy blood donors in Brazil, a country considered an ethnic melting pot. The translocation was detected by nested PCR in 227 peripheral blood samples from individuals with different ethnic backgrounds. The t(14;18) translocation was found in 45 of 85 White individuals (52.94%); in 57 of 72 Black individuals (79.17%); and in 68 of 70 individuals (97.14%) of Japanese-descent. In conclusion, the frequency of the t(14;18) translocation in the Brazilian population varies according to the ethnic background.

## Introduction

The chromosomal translocation involving the long arms of chromosomes 14 and 18 has been detected cytogenetically in about 90% of follicular lymphomas and 20–30% of diffuse large cell lymphomas (1). The t(14;18) translocation involves the *bcl2* gene, which is translocated into the immunoglobulin heavy (IgH) chain locus (2). As a result, *bcl2* expression is controlled by the promoter of the constitutive IgH leading to high transcription levels of this chimeric RNA. The coding sequence of the *bcl2* transcript is not altered and the resulting protein is identical to that from the normal gene (3). Nearly 60% of the t(14;18)-translocations are clustered within a 166 bp sequence of the so-called major breakpoint region (MBR) (4).

The unusually high levels of *bcl2* create t(14;18)-positive B lymphocytes that can accumulate and escape from their natural control mechanism. The t(14;18) translocation is not sufficient for lymphoma development, as demonstrated by the presence of t(14;18)-positive lymphocytes in healthy individuals (5). This translocation is the same found in follicular lymphoma (1). More than 50% of Western European and North American healthy individuals have circulating B-cells that carry this translocation (2). Nevertheless, the percentage of healthy individuals carrying the t(14;18) translocation varies greatly among different populations (1,5,6).

There are significant differences in the frequency of the t(14;18) translocation in populations from different countries. To the best of our knowledge, there are no studies in Black populations. Brazil is a country with a known ethnic diversity, which allows analyses of different genetic profiles. The frequency of the t(14;18) translocation was 74% in patients with follicular lymphoma when determined by fluorescence *in situ* hybridization (FISH) (7). However, no data on the Brazilian healthy population have been reported. Here, we describe the frequency of the t(14;18) translocation in a Brazilian population of healthy individuals with different ethnic backgrounds.

## Material and Methods

### Population samples

A total of 227 peripheral blood samples were collected from subjects ranging from 18 to 71 years old. The samples were collected from healthy blood donors from Fundação Pró-Sangue Hemocentro de São Paulo, after they signed a written informed consent form, according to the protocol approved by the Ethics Committee on Human Research of the institutions. Subjects were asked about their ethnicity as previously described by our group (8).

### DNA isolation and nested PCR

DNA from peripheral blood mononuclear cells was extracted from 500 µL of whole human blood samples using a salting out method with slight modifications, as previously described (9). DNA from a follicular lymphoma patient was used as a positive control. Karpas-422 cell dilutions were used to calculate the PCR detection limit. For nested PCR, the MBR of the t(14;18) translocation was amplified by a two-step nested PCR as previously described (10).

The following pairs of oligonucleotides (Integrated DNA Technologies, USA) were used: first step – sense: 5′-GAC CAG CAG ATT CAA ATC TAT GGT GGT-3′; antisense: 5′-GGA CTC ACC TGA GGA GAC GGT G -3′; second step - sense: 5′-CCT TTA GAG AGT TGC TTT ACG TGG CC-3′; antisense: 5′-GGA GAC GGT GAC CAG GGT-3′.

The first step of PCR amplification was performed in a 50-µL reaction mixture containing 500 ng genomic DNA, 0.36 μM of each first step primer, 0.2 mM dNTP, 1.50 mM MgCl, 50 mM KCl, 10 mM Tris-HCl, pH 8.3, and 0.5 U Taq DNA polymerase using a PT100 JM Research thermocycler. Conditions were 25 cycles at 95°C for 30 s, 54°C for 40 s, 72°C for 45 s.

**Figure 1.**
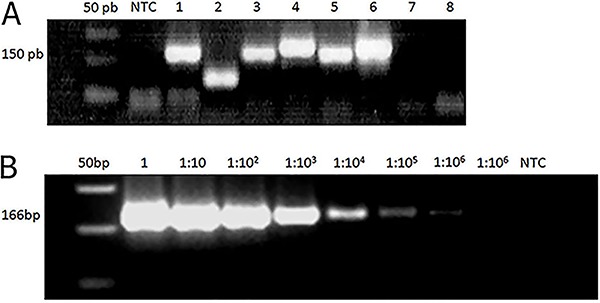
Nested PCR of bcl-2/IGH rearrangement. *A*, Amplification products of NTC (no template control); 1–6: positive subjects; 7 and 8: negative subjects. *B*, Detection limits of *bcl2*/IgH rearrangement test. Karpas-422 cells were increasingly diluted with bcl2/IGH negative cells. Detection limit was 1:10^6^ dilution.

The second step was performed in a 50-µL reaction mixture containing 3 µL of the PCR product obtained in the first reaction, 0.48 μM of each second step primer, 0.2 mM each dNTP, 1.50 mM MgCl, 50 mM KCl, 10 mM Tris-HCl, pH 8.3, and 0.5 U Taq DNA polymerase. Thermocycler conditions were 30 cycles at 95°C for 30 s, 55°C for 40 s, 72°C for 45 s. Amplification products were analyzed by electrophoresis on 3% (w/v) agarose gel after staining with gel red (Figure 1A).

Karpas-422 cell dilutions were used to test the detection limit with a reproducible detection of one t(14;18) copy in 105 (1:100,000) cells and a maximum detection limit of 1:106 (1:1,000,000) cells (Figure 1B). The detection limits were very similar to those described by Schmitt el al. (10) confirming the robustness of this nested PCR.

### Statistical analysis

The X^2^-test was used to evaluate differences between groups (Software: GraphPad Prism 5, USA).

## Results and Discussion

Studies have associated follicular lymphoma risk factors with the prevalence and/or frequency of t(14;18)-positive cells in individuals without lymphoma (3). The presence of the BCL2/IgH rearrangement in the peripheral blood of healthy individuals is well documented (3,11). In this study, the frequency of the t(14;18)-MBR translocation was determined in 227 DNA samples from normal individuals using nested PCR.

Brazilians are one of the most heterogeneous populations in the world due to extensive admixture of three different ancestral roots: American Indians, Europeans, and Africans (12). Japanese immigration is more recent. The Brazilian population is 62.6% White, 5.8% Black, 30.8% Mulatto, and 0.8% Japanese-descendants (8). The ethnic composition of the subjects in this study is summarized in Table 1. Eighty-five individuals were White (median age: 45 years; 43.53% male), 72 were Black (median age: 45 years; 50.00% male), and 70 were Japanese-descendants (median age: 34.5 years; 47.14% male).

**Table 1.** Age and gender of the individuals evaluated for the t(14;18) translocation, according to ethnic group.

	Age		Gender	
	Median	Range	Male	Female
White (n=85)	45.0	19–71	37 (43.53%)	48 (56.47%)
Black (n=72)	32.5	19–56	54 (75.00%)	18 (25.00%)
Japanese descendant (n=70)	34.5	18–63	33 (47.14%)	37 (52.86%)

There are large discrepancies in the frequencies of the BCL2/IgH translocation reported in healthy subjects, ranging from 8 to 88%, according to Schüler et al. (2) The variability between studies is mainly due to the sensitivity of the technique, the initial concentration of DNA, and the tissue origin (2,13). However, there are factors not related to experimental variations, such as the age of the individual (10), exposure to pesticides (14), the family history of hematopoietic diseases (14), smoking (3), and others.

**Figure 2.**
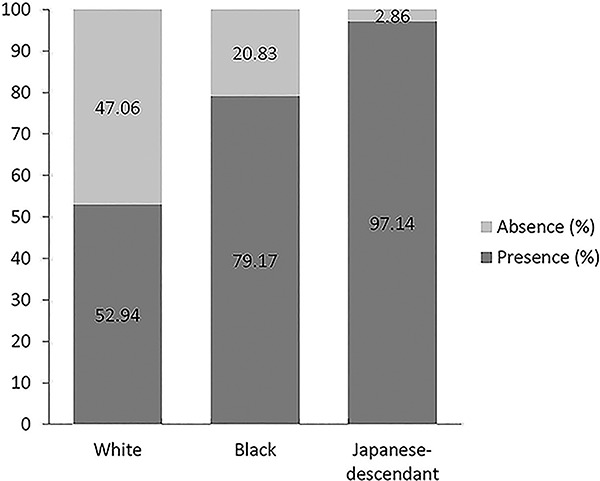
Frequency of t(14;18) translocation in different ethnic groups, as determined by nested-PCR. The X^2^-test was used to demonstrate differences between groups.

In our study, the t(14;18)-MBR translocation frequency varied between the ethnic groups. The translocation was found in 52.94% White individuals, 79.17% Black individuals, and in 97.14% of the Japanese-descendants (Figure 2). The frequency in White subjects was similar to values previously reported. This is the first description of the t(14;18)-MBR translocation frequency in a Black population, and the frequency was higher than in the White population. One unexpected observation from our study was the high frequency of the translocation in Japanese-descendants. Few studies have been performed in Japanese populations. A low frequency (15.45%) of the BCL2/IgH translocation was detected in peripheral blood lymphocytes of healthy Japanese individuals (15). Another study of Chinese individuals of the Han nationality, located in the Zhejiang area, reported a translocation frequency of 9.66% (6). The Japanese-descendant population in Brazil is mostly from Okinawa Island. The population of this island represents 1% of Japan's population. There are no data available for the population of Okinawa. Nevertheless, the difference in the frequency found in this study is likely to be due to geographical segregation.

In conclusion, we demonstrated that the frequency of the t(14;18)-MBR translocation in Brazil varied according to the individual's ethnic origin. The highest (97.14%) frequency was found in Japanese descendants, while an intermediate frequency (79.17%) was detected in the Black population and the lowest frequency (52.94%) in the White population. Therefore, this translocation is not considered a good marker for follicular lymphoma in Brazil.
